# Synthesis and Evaluation of Novel 2-((1*H*-1,2,4-triazol-5-yl)thio)-*N*-benzylidene-*N*-phenylacetohydrazide as Potential Antimicrobial Agents

**DOI:** 10.3390/ijms262412078

**Published:** 2025-12-16

**Authors:** Athul S., Bhuvaneshwari S. V., Avani Anu G., Parvathi Mohanan P. C., Anu R. Melge, Aravind Madhavan, Bipin G. Nair, Geetha Kumar, Vipin A. Nair, Pradeesh Babu

**Affiliations:** 1 Amrita School of Biotechnology, Amrita Vishwa Vidyapeetham, Kollam 690525, Kerala, India; 2 Rukmini Educational Trust, REVA University, Bengaluru 560064, Karnataka, India

**Keywords:** 2-((1*H*-1,2,4-triazol-5-yl)thio)-*N*-benzylidene-*N*-arylacetohydrazide hybrid, SwissADME, antibacterial agents, antimicrobial resistance

## Abstract

This study details the design, synthesis, and evaluation of a novel series of fourteen 2-((1*H*-1,2,4-triazol-5-yl)thio)-*N*-benzylidene-*N*-arylacetohydrazide hybrid compounds. The primary objective was to investigate their potential as antimicrobial agents and assess their cytotoxicity. A systematic approach combining in silico screening and experimental validation was employed. The initial in silico analysis, using SwissADME, identified compounds with favorable drug-like properties. Subsequently, all fourteen compounds were synthesized and characterized using various spectroscopic methods. Their antibacterial efficacy was evaluated in vitro against Gram-negative (*Klebsiella aerogenes*) and Gram-positive (*Enterococcus* sp.) bacteria through growth kinetics and colony-forming unit (CFU) assays. Cytotoxicity was assessed using MTT assays on HEK (human embryonic kidney) cell lines. The compound, 2-((1*H*-1,2,4-triazol-3-yl)thio)-*N′-*(2-fluorobenzylidene)-*N*-phenylacetohydrazide emerged as the most promising candidate, demonstrating broad-spectrum antibacterial activity. These findings highlight the potential of 2-((1*H*-1,2,4-triazol-5-yl)thio)-*N*-benzylidene-*N*-arylacetohydrazide hybrids as a scaffold for developing new antimicrobial agents. Furthermore, this study suggests possible environmental applications for these compounds in antimicrobial resistance (AMR) management.

## 1. Introduction

**Figure 1:**
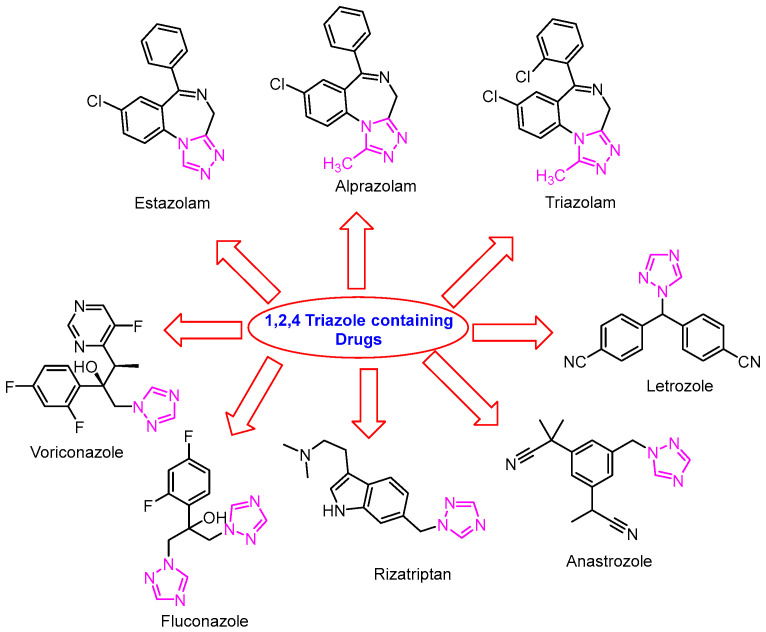
Examples of commercial drugs containing the 1,2,4-triazole ring.

1,2,4-Triazoles are stable, five-membered aromatic heterocycles that are composed of two carbon atoms and three nitrogen atoms [1]. These compounds exist in two possible isomeric forms, depending on the nitrogen atom arrangement, and exhibit tautomerism based on the nitrogen bonded to hydrogen [2]. Due to their high solubility, structural rigidity, chemical stability, and favorable dipole moment, 1,2,4-triazoles serve as versatile scaffolds, with significant applications in medicinal chemistry, materials science, and polymer development [3,4,5]. Many triazole-based compounds have been successfully incorporated into marketed pharmaceuticals, including antiviral [6], antifungal [7], anti-anxiety, and anticancer agents [8], (Figure 1) demonstrating their broad therapeutic utility.

S-alkylated triazole-3-thiols derived from methoxy-substituted phenyl groups demonstrate strong anti-inflammatory effects [9], while other S-substituted triazole derivatives exhibit broad antibacterial activity [10]. Certain triazole-thione compounds have a high affinity for serotonin receptors, suggesting potential applications in neuropharmacology [11]. Additionally, some triazole-based heterocycles effectively chelate toxic heavy metals, offering benefits for both environmental cleanup and biological use [12]. Given the significant roles of S-triazoles in medicine and the environment [13], there is an urgent need to develop faster and simpler synthetic methods to expand their applications efficiently. Based on our interest in the applications of heterocyclic compounds [14,15,16,17,18,19], we decided to synthesize 2-((1*H*-1,2,4-triazol-5-yl)thio)-*N*-benzylidene-*N*-arylacetohydrazide and explore its biological properties.

Antimicrobial resistance (AMR) is a growing crisis that is caused by both Gram-negative and Gram-positive bacteria. These bacteria develop sophisticated mechanisms to evade antibiotics. Gram-negative bacteria are often more resistant due to their additional outer membrane. This blocks antibiotic entry and enables effective use of efflux pumps. This leads to the rapid modification or destruction of antibiotics through enzymes like β-lactamases. They often cause severe healthcare-associated infections and have high rates of resistance [20]. Gram-positive bacteria lack that outer membrane. However, they are also good at developing resistance via enzymatic degradation (e.g., β-lactamases) and modifying antibiotic binding sites [21]. This is facilitated by acquiring or altering the genes that are responsible for target proteins [22].

Identification and development of new chemical entities (NCEs) is a promising alternate strategy against AMR. NCEs are small, novel structures and mechanism-of-action compounds. They are different from the modification of existing antibiotics in that they can interact with bacterial processes in new ways. They can inhibit biofilm formation or block crucial biosynthetic pathways. This prevents the rapid development of resistance by the bacteria [23]. The primary strengths of NCEs are their ability for structural diversity, various biological activities, and biocompatibility. This renders them excellent candidates for overcoming the current limitations of antibiotics and contributing to the global war against resistant infections.

Research underscores the sustainable discovery and strategic design of NCEs with improved mechanisms for screening, development, and regulation. These are the crucial steps toward effectively addressing the escalating threat of antimicrobial resistance [24]. Heterocyclic compounds, particularly those incorporating nitrogen and sulfur atoms, have long been indispensable scaffolds in medicinal chemistry. They serve as the core structure for numerous drugs with broad-spectrum biological activities. Among these, the 1,2,4-triazole nucleus is widely recognized as a privileged structure. This has been frequently documented in compounds exhibiting significant antimicrobial, antiviral, and antifungal properties [25,26,27]. The synthesis and antibacterial study of 3-[1-(4-fluorobenzyl)-1*H*-indol-3-yl]-5-(4-fluorobenzylthio)-4*H*-1,2,4-triazole-4-amine and its Schiff bases confirmed that the aromatic substituent at the 4-position of the triazole ring plays a crucial role in antibacterial activity. Particularly, the presence of halogen and nitrogen groups significantly enhanced the inhibitory activity against all tested bacteria. This improves the interaction with bacterial targets and membrane penetration, increasing antibacterial potency [26]. *N*-Cyclohexyl-2-[5-(4-pyridyl)-4-(*p*-tolyl)-4*H*-1,2,4-triazole-3-sulfanyl]-acetamide was synthesized to evaluate antibacterial activity. This compound demonstrated a good level of inhibition, comparable to ciprofloxacin [25]. The structural versatility of the triazole ring allows for diverse chemical modifications, such as fusion with other bioactive rings, like indole, or substitution with thiolated functionalities [28]. Indole derivatives of 4-amino-4*H*-1,2,4-triazole-3-thiol showed antibacterial activity against four pathogenic strains. Studies revealed that the amino-containing derivatives had a highly potent inhibitory effect against *S. aureus* and *E. coli* than amoxicillin. The acetohydrazide compound, 2-{3-[4-bromobenzylidene)amino]-1*H*-1,2,4-triazol-5-ylthio}acetohydrazide, exhibited strong antibacterial activity against the tested *S. aureus* and *E. coli*. This activity level was comparable to ampicillin. This indicates that the acetohydrazide derivative is a potent antibacterial agent, demonstrating efficacy that is close to that of the standard antibiotic ampicillin [28]. This sustained focus on the 1,2,4-triazole system underscores its critical importance in the search for effective agents to combat resistant microbial strains.

Modern drug design often involves hybridizing known bioactive scaffolds to enhance potency and introduce novel mechanisms of action. This strategy has been successfully applied to optimize existing drugs, since this class of compounds has an established pharmacological profile. Such an example is the design and synthesis of novel ofloxacin analogs to overcome resistance limitations [29]. These derivatives have antibacterial activity against all tested Gram-positive bacteria and one Gram-negative pathogen. The antibacterial properties of these derivatives were found to be comparable to those of ofloxacin. Specifically, compounds featuring a thiol-linked 1,2,4-triazole moiety are of particular interest due to the increased biological relevance imparted by the sulfur atom.

In this study, we synthesized a novel series of 2-((1*H*-1,2,4-triazol-5-yl)thio)-*N*-benzylidene-*N*-arylacetohydrazide hybrids (**4a**–**4n**) and evaluated their efficacy against a panel of both Gram-positive and Gram-negative bacterial strains to assess their broad-spectrum activity. These compounds have potency in demonstrating potent antibacterial activity against drug-resistant pathogens, making them promising candidates for new antimicrobial agents [30]. The findings from this investigation will provide crucial insights into the antibacterial potential of these compounds, positioning them as promising lead compounds for future antibiotic development.

## 2. Results

### 2.1. Chemistry

**Scheme 1:**
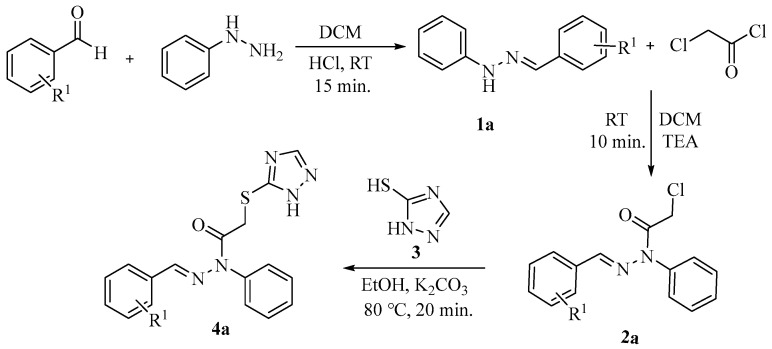
Synthesis of 2-((1*H*-1,2,4-triazol-5-yl)thio)-*N*-substituted benzylidene-*N*-phenylacetohydrazide derivatives.

This study focuses on synthesizing the novel compound 2-((1*H*-1,2,4-triazol-5-yl)thio)-*N*-benzylidene-*N*-arylacetohydrazide (**4a**–**4n**) via nucleophilic substitution, as outlined in Scheme 1. Commercially available 1*H*-1,2,4-triazole-3-thiol (**3**) and Schiff base (**1a**) were used as key intermediates. The synthetic route, illustrated in Scheme 1, involved multiple steps. First, 1-benzylidene-2-phenylhydrazine (**1a**) was prepared by reacting an aldehyde (A) with phenyl hydrazine (B). Further, *N*-benzylidene-2-chloro-*N*-phenylacetohydrazide (**2a**) was synthesized by treating 1a with 2-chloroacetyl chloride [31,32,33], and finally, (**2a**) was then subjected to nucleophilic substitution with 1,2,4-triazole-3-thiol (**3**) in the presence of potassium carbonate (K_2_CO_3_) as a base, yielding the target compound **4a** (Scheme 1). This method employs green chemistry principles, using inexpensive reagents and mild conditions to efficiently produce novel triazole derivatives.

To prepare 2-((1*H*-1,2,4-triazol-5-yl)thio)-*N*-benzylidene-*N*-phenylacetohydrazide derivatives, we optimized key reaction parameters, including the solvent choice, base, temperature, reaction time, and yield. Initial trials utilized potassium carbonate (K_2_CO_3_) as the base while screening various solvents (Table 1, Entries 1–9). Non-polar solvents like toluene gave modest yields of up to 55%, whereas ether-based solvents such as THF and 1,4-dioxane delivered moderate yields but required longer reaction times of around 3 h. Polar aprotic solvents, such as acetonitrile, improved the yield to 74% within 2 h. Further enhancements were achieved using higher boiling polar solvents like DMF and DMSO, which raised the yields to 75–78% and reduced the reaction times to approximately 1.5 h, suggesting a positive correlation between solvent polarity and reaction efficiency. Extending the investigation to polar protic solvents, which typically favor nucleophilic substitutions, revealed significant improvements; isopropanol and methanol increased the yields to 80% and 84%, respectively. Notably, ethanol proved the most effective, enabling a nearly complete conversion, with yields of up to 90% in just 20 min (Table 1, Entry 13). Consequently, EtOH was selected for further optimization, involving variations in base and temperature.

To further explore the substrate scope, the model reaction was evaluated using various bases (Table 1, Entries 10–14). Overall, all tested bases provided moderate to good yields, with the inorganic bases outperforming their organic counterparts. The organic bases triethylamine (TEA) and diisopropylethylamine (DIPEA) gave yields of 65% and 68% after 2 h, respectively. In comparison, inorganic weak bases, such as sodium bicarbonate (NaHCO_3_) and sodium carbonate (Na_2_CO_3_), significantly improved the yields to 83% and 80% respectively, while reducing the reaction time to under one hour. Most notably, potassium carbonate (K_2_CO_3_), a cost-effective and non-nucleophilic inorganic base, delivered the highest yield of 90% in just 20 min. Based on these results, K_2_CO_3_ was chosen as the optimal base for subsequent experiments to maximize reaction efficiency. At room temperature, the reaction conversion was limited to around 50%. Raising the temperature to 50 °C markedly accelerated the reaction, reducing the required time by 2 h. Further increasing the temperature to 80 °C caused a dramatic boost in the reaction rate, reducing the reaction time from 3 h to just 20 min and achieving a maximum yield of approximately 90% (Table 1, Entry 13). Under these optimized conditions, using K_2_CO_3_ as the base in ethanol at a reflux temperature of 80 °C, the 2-((1*H*-1,2,4-triazol-5-yl)thio)-*N*-arylidene-*N*-phenylacetohydrazide (**4a**–**4n**) were synthesized efficiently with high yield (Table 2). All the synthesized compounds were characterized by ^1^H & ^13^C NMR and high-resolution mass spectral analysis. In the ^13^C NMR spectra, carbon-fluorine spin–spin couplings were observed, with coupling constants of 2.4 Hz, 8.2 Hz for the compound **4d**, and 3.0 Hz for the compound **4g**.

### 2.2. Evaluation of Physicochemical Characteristics

All the compounds (**4a**–**4n**) comply with Lipinski’s rule. This suggests that the compounds are more likely to have good oral bioavailability, and zero PAIN alerts indicate the drug-like properties (Table S1) [34]. All these synthesized 2-((1*H*-1,2,4-triazol-5-yl)thio)-*N*-benzylidene-*N*-phenylacetohydrazide derivatives hav’e <500, <5, <10, and <150 A^2^, respectively, for the molecular weight (MW), hydrogen bond donor (HBD), hydrogen bond acceptor (HBA), and topological polar surface area (TPSA). The compounds **4a** to **4j** have the same F. Csp^3^ of 0.06. The remainder of the compounds (**4k** to **4n**) have the values 0.11, 0.20, 0.16, and 0.07, respectively. All these compounds exhibited ≤ 10 rotatable bonds (RB) and a molar refractivity within the range of 90 and 115 [35].

Brenk alerts are warning structural alerts that indicate potentially toxic or problematic substructures within a molecule. Among the datasets generated from the compounds, all except **4f** had only a single Brenk alert, while **4f** showed three alerts. This can be an indication of the reactive or offending moieties that can affect the safety or stability. The compounds’ lead-likeness violations were within **1** and **3**, indicating that these compounds might be outside the optimal range for lead compounds.

The compounds have moderate lipophilicity (iLOGP ~ 2-3), which is ideal for balancing membrane permeability and solubility (Table S1). No compounds were excessively lipophilic (>5), which reduces the risk of bioaccumulation and toxicity [36]. Most compounds show very low solubility, ranging from 0.0001 to 0.0005 mg/mL (Table S1). This is a concern, as poor solubility can severely impact oral bioavailability. Compounds with higher solubility are generally easier to formulate into oral tablets or capsules [37].

**Figure 2:**
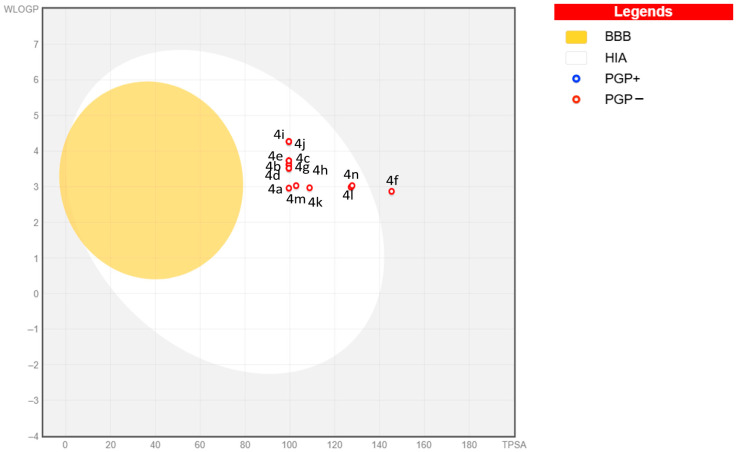
The boiled egg plot of compounds plotted with the SwissADME database. Compounds in egg white: **4a**, **4b**, **4c**, **4d**, **4e**, **4g**, **4h**, **4i**, **4j**, **4k**, **4l**, **4m**, and **4n**. Compounds outside boiled egg representation: **4f**.

**Figure 3:**
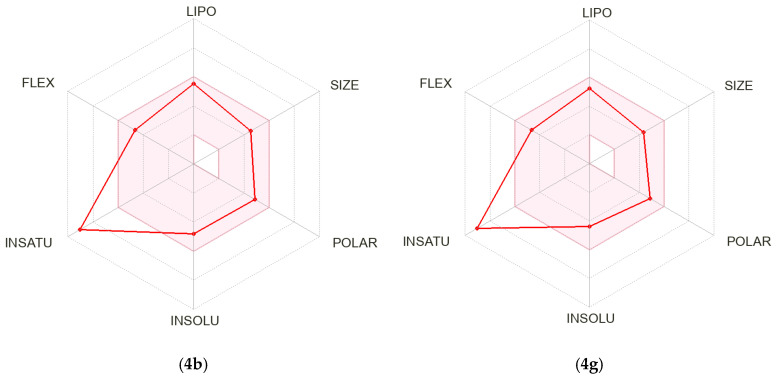

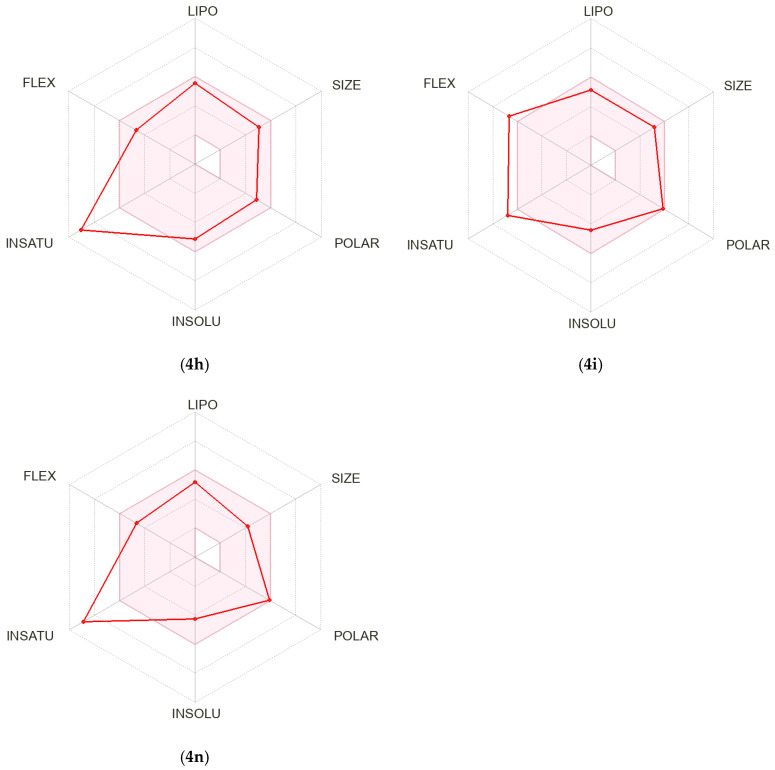
The radar charts of compounds (**4b**, **4g**, **4h**, **4l**, and **4n**) based on the physicochemical properties. Radar plot of important physicochemical attributes of a compound. The axes point to six molecular descriptors as follows: LIPO (lipophilicity), SIZE (size of the molecule), POLAR (polarity), INSOLU (insolubility or index related to solubility), INSATU (unsaturation index), and FLEX (molecular flexibility). The red contour indicates the compound’s profile over these properties, and the gray-filled area reflects the ideal range for drug-like molecules, useful in the determination of drug-likeness and bioavailability.

Pharmacokinetics is essential for achieving the desired pharmacological effect of a drug on a healthy person. This indicates that each compound’s pharmacokinetic property can potentially influence a drug’s pharmacological profile. All compounds were predicted to have high GI absorption, except for **4f**, according to the SwissADME database (Table S1). Figure 2 indicates the boiled egg depiction of the compounds. The plot uses a graph with WLOGP (water-octanol partition coefficient) on the x-axis and TPSA (topological polar surface area) on the y-axis. The compounds, except **4f**, are likely to have high intestinal absorption since they are positioned in the “egg white”. However, these compounds are not permeable across the blood–brain barrier, and the plot of these compounds clearly indicates the result (Figure 2). The compounds have no effect on the p-glycoprotein (Pgp) efflux pump since they are not a substrate for Pgp. In the case of drug metabolism, the compounds are unaffected by CYP2D6 inhibitors. Compounds **4i**, **4k**, **4l**, and **4m** are CYP3A4 inhibitors, while the rest are unaffected. All the compounds exhibited a bioavailability score of 0.55. Table S1 also depicts the five drug-likeness approaches (Lipinski’s, Ghose, Veber, Egan, and Muegge) that are not violated by this series of compounds. The important physiochemical attributes of the compounds **4b**, **4g**, **4h**, **4l** and **4n** (Figure 3) are depicted using a radar plot.

### 2.3. Preliminary Screening of Antibacterial Activity

**Figure 4:**
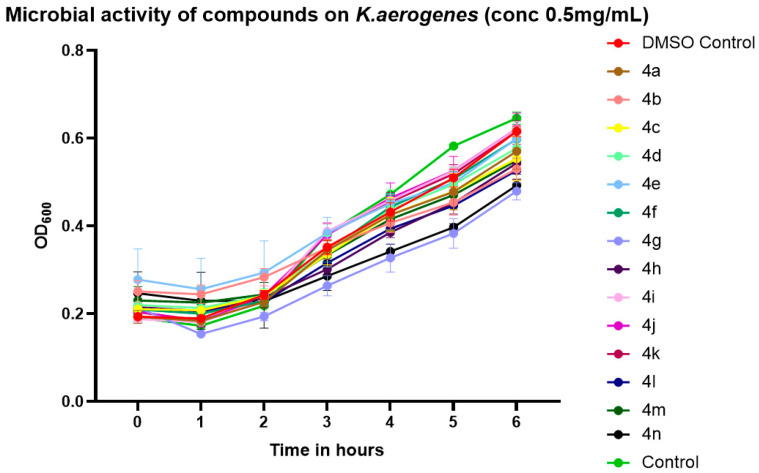
The graph shows the antimicrobial activity of compounds (concentration 0.5 mg/mL) against *K. aerogenes*.

**Figure 5:**
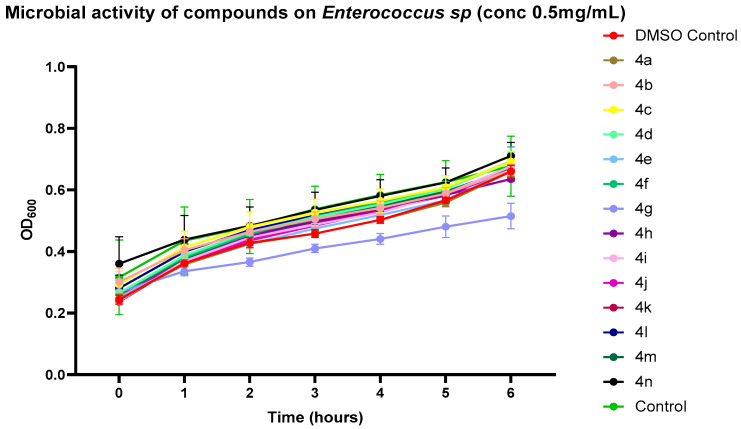
The graph shows the antimicrobial activity of compounds (concentration 0.5 mg/mL) against *Enterococcus* sp.

The antimicrobial efficacy of the test compounds (**4a**–**4n**) was determined using growth kinetics assays against *K. aerogenes* and *Enterococcus* sp. at a concentration of 0.5 mg/mL. The *ATCC 13048* untreated control and DMSO control were both showing consistent growth, reaching an OD_600_ of ~0.68 at 6 h. Of the compounds, **4g** and **4n** expressed the most significant growth inhibition by low OD_600_ readings throughout the course. Furthermore, **4b**, **4h**, and **4l** expressed mild inhibitory activities, while the remainder of the compounds showed virtually identical growth kinetics to the untreated control and, thus, had minimal efficacy (Figure 4). However, *MV BP 18* differed from this pattern of susceptibility. These were followed by **4g**, which was the most potent inhibitor, with low OD_600_ readings throughout the 6 h time course, whereas most of the other compounds were not significantly effective in inhibiting bacterial growth (Figure 5). These results suggest some degree of strain-specific antimicrobial activity in the tested compounds, with **4g** being the broadest spectrum of activity among the two tested strains. Such an observation acts to further emphasize the potential of specific antimicrobial approaches and the promise of **4b**, **4g**, **4h**, **4l,** and **4n** as candidate compounds for further study.

### 2.4. Validation of Antibacterial Activity Through Growth Kinetics and CFU Enumeration

**Figure 6:**
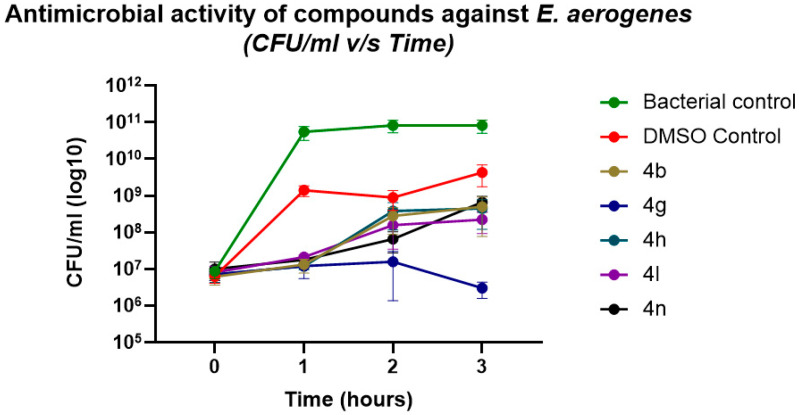
Time–kill assay showing the antimicrobial activity of compounds against *K. aerogenes*, represented as viable cell counts (CFU/mL) over time. Data are presented as mean ± standard deviation from triplicate experiments.

The antimicrobial action of compounds (**4a**–**4n**) against *K. aerogenes* was measured by colony-forming unit (CFU/mL) assays over time. The findings by CFU counting (Figure 6) provided us with a quantification of the number of viable bacterial cells over time. The DMSO control bacteria grew extremely rapidly from ~10^7^ to ~10^9^ CFU/mL within 1 h and remained at this high rate through 3 h. Compound **4g** again performed outstandingly, significantly reducing viable counts to below 10^7^ CFU/mL at 2 h and maintaining it at 3 h, affirming its potent bacterial activity. Compounds **4l** and **4n** had a moderate-level reduction in the bacterial count, with terminal values averaging 10^8^–10^9^ CFU/mL. Compounds **4b** and **4h** caused partial inhibition of growth, but to no lesser extent than **4g**.

**Figure 7:**
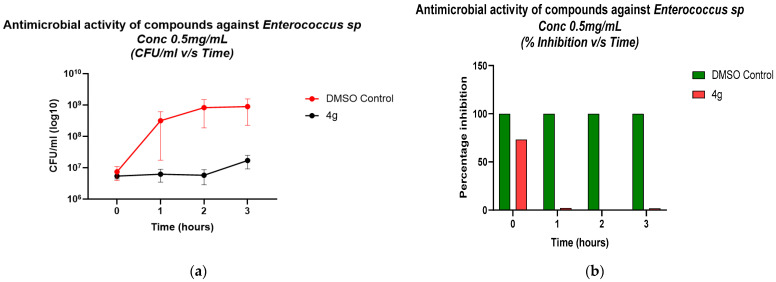
(**a**) Time–kill assay showing the antimicrobial activity of compounds against Enterococcus sp., represented as viable cell counts (CFU/mL) over time. Data are presented as mean ± standard deviation from triplicate experiments. (**b**) Percentage inhibition v/s. time graph showing the antimicrobial activity of ‘**4g**’ with respect to the DMSO control.

The antimicrobial efficacy of **4g** against *Enterococcus* sp. was evaluated using colony-forming unit (CFU/mL) assays across a time course. The findings revealed that **4g** treatment led to a reduction in viable bacterial counts over the 3 h incubation period. While the DMSO control rapidly increased from ~10^6^ to ~10^8^ CFU/mL by 1 h and plateaued thereafter, **4g** maintained bacterial counts near baseline (~10^7^ CFU/mL), with only a slight increase by hour 3 (Figure 7a). This consistency between OD_600_ and CFU/mL data reinforces the compound’s bacteriostatic and possibly bactericidal potential. Figure 7b illustrates the antimicrobial activity of compound **4g** compared to the control DMSO against the organism Enterococcus sp., expressed as a percentage inhibition over time. While the control was always strongly inhibited, compound **4g** only exhibited partial inhibition at 0 h and thereafter totally lost activity.

### 2.5. Cytotoxicity Assessment of Compounds Using MTT Assay HEK Cell Lines

**Figure 8:**
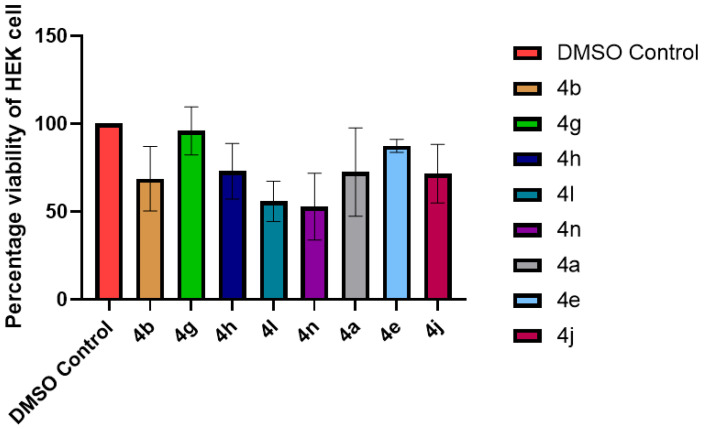
Cytotoxicity analysis of compounds on HEK cells using MTT assay. Bar graph showing the percentage viability of HEK (human embryonic kidney) cells following treatment with compounds. The results suggest that certain compounds may exert selective cytotoxicity, with reduced toxicity toward non-cancerous human cells. Data are expressed as mean ± SEM.

To determine the cytotoxicity of test compounds toward HEK cells, cell viability was assayed at a concentration of 0.5mg/mL, and the percentage viability compared to the DMSO control was determined (Figure 8). As such, 100.00% cell viability for the DMSO control proved to be the baseline viability in the absence of cytotoxic agents. Of the compounds under test, **4g** showed the best cell viability at 95.96%, which was very close to the DMSO control, which reflected little cytotoxicity. This was followed by **4e** (87.41%), reflecting the same low degree of cytotoxic effects. Compounds **4h** (73.03%), **4a** (72.56%), and **4j** (71.60%) also had moderately high cell viability, although with slightly higher cytotoxicity than **4g** and **4e**. Conversely, compounds **4b** (68.67%), **4l** (55.83%), and **4n** (52.76%) had a greater decrease in HEK cell viability, showing relatively greater cytotoxicity. Of all, **4n** had the lowest viability at 52.76%, indicating strong cytotoxic potential. These findings collectively imply that compounds **4g** and **4e** have the greatest biocompatibility towards HEK cells, but **4n** and **4l** showed greater cytotoxicity at the 0.5 mg/mL concentration.

## 3. Discussion

In this study, a series of novel 2-((1*H*-1,2,4-triazol-5-yl)thio)-*N*-benzylidene-*N*-phenylacetohydrazide (**4a**–**4n**) were synthesized, and their therapeutic potential was evaluated. Researchers continue to focus on the 1,2,4-triazole derivatives due to their wide range of medicinal applications. Incorporation of a mercapto group into the 1,2,4-triazole ring significantly enhances its biological activity [38]. In particular, 1,2,4-triazole-3-thiols have gained increasing attention for their notable cytotoxic and antimicrobial properties, making them significantly valuable in medicine. They also hold importance in agriculture [39], industry [40], analytical chemistry, and photographic processes [41]. These compounds serve as versatile intermediates for numerous syntheses, showing promise not only in cancer treatment research but also in antimicrobial drug development [42]. In the current study, we have developed an efficient and practical synthesis of novel 2-((1*H*-1,2,4-triazol-5-yl)thio)-*N*-benzylidene-*N*-phenylacetohydrazide and its derivatives, obtained in good yields and subsequently subjected to antimicrobial screening.

The molecular design integrated two well-known antimicrobial pharmacophores, aiming to create potent new scaffolds. The initial in silico screening was a crucial first step. The compounds’ adherence to Lipinski’s rules and lack of PAINs alerts (Table S1) suggested their feasibility as drug candidates [34,43]. In vitro antibacterial activity using growth kinetics showed compound **4g** to have the highest degree of growth inhibition compared to *K. aerogenes* (ATCC 13048), followed by **4h**, **4l**, and **4n** (Figure 4). The growth curves demonstrated sustained suppression in OD_600_ up to 6 h, reflective of either a bacteriostatic effect or a bactericidal effect. These trends were also observed in CFU enumeration tests, where **4g** reduced viable numbers by several orders of magnitude within 3 h (Figure 6). These observations take on an even greater significance in the context of *K. aerogenes’* position in the *ESKAPE* pathogens, which are particularly known for their biofilm development and multidrug resistance [44,45].

Interestingly, **4g** was also active against *Enterococcus* sp. (MV BP 18), a Gram-positive bacterium, further confirming its broad-spectrum potential (Figure 5 and Figure 7). Furthermore, **4g**’s double activity against both Gram-negative and Gram-positive bacteria was remarkable, especially considering the intrinsic differences in cell wall structures and efflux mechanisms that typically hamper cross-activity [46,47]. In contrast, other molecules like **4b** and **4l** were strain-specifically active, highlighting the necessity of personalized antibacterial strategies and structure-activity relationship (SAR) optimization.

A critical aspect of any potential therapeutic is its safety profile. The cytotoxicity assays provided valuable insights, revealing that several compounds were less toxic to the HEK cell line. Compound **4g**, in particular, showed about 95% cell viability as compared with the DMSO control, indicating its less toxic nature (Figure 8). While the results are promising, this study also identified challenges. The low aqueous solubility of the compounds is a concern for bioavailability and will require formulation strategies to overcome (Table S1). Additionally, the in silico prediction of potential CYP450 inhibition by **4g** indicates that further optimization and pharmacokinetic studies are necessary to improve its safety profile (Table S1).

Beyond clinical applications, the properties of these compounds suggest potential for environmental use. With the rise of antimicrobial resistance as a “One Health” issue spanning human, animal, and environmental health, novel compounds are needed to control the spread of resistant pathogens in settings like wastewater treatment plants and agricultural runoff [48,49,50]. The broad-spectrum efficacy, stability, and low solubility of these compounds could make them suitable for use in antimicrobial coatings, filters, or disinfection systems, offering a slow-release mechanism to decontaminate high-risk environments.

## 4. Materials and Methods

Commercially available chemicals were used without purification. Reactions were conducted in oven-dried glassware under controlled atmospheric conditions. The melting points were determined on a Melt-temp apparatus and were uncorrected. TLC was performed on Merck silica gel 60 F254 (Merck KgaA, Darmstadt, Germany) using n-hexane/ethyl acetate as eluents, and the spots were visualized by UV light. ^1^H NMR spectra were recorded on an Avance Bruker NMR spectrometer at 400MHz (Merck KgaA, Darmstadt, Germany), and the ^13^C NMR spectra were recorded on the same instrument at 100 MHz using TMS as the internal standard. Chemical shifts were expressed in parts per million (ppm). High-resolution mass spectrometric analysis was carried out in a Waters-Xevo G2-XS-QToF mass spectrometer (Merck KgaA, Darmstadt, Germany, Milford, CT, USA).

### 4.1. Procedure for Synthesis of 1-Benzylidene-2-phenylhydrazine (***1a***–***1n***)

To a solution of benzaldehyde (0.11 g, 1.0 mmol) in EtOH (10 mL), cat. HCl was added and stirred at room temperature. To this solution, Phenyl hydrazine (0.11 g, 1.0 mmol) was added, and the reaction mixture was stirred at RT for 15 min. The progress of the reaction was monitored by TLC, and ice-cold water was added. The formed precipitate in the reaction mixture was filtered and washed with hexane and then dried. The crude product was subjected to further steps without purification.

### 4.2. Procedure for Synthesis of E-1-Benzylidene-2-chloro phenylacetohydrazide (***2a***–***2n***)

To a cooled solution of 1-benzylidene-2-phenylhydrazine (0.2 g, 1.0 mmol) in DCM (10 mL), TEA (0.1 g, 1.0 mmol) was added. To this reaction mixture, 2-chloroacetyl chloride (0.11 g, 1.0 mmol) was added and stirred at RT for 10 min. The progress of the reaction was monitored by TLC, the solvent was evaporated under reduced pressure, and ice-cold water was added to the obtained solid and filtered, washed with hexane, and then dried. The crude product was subjected to further steps without purification.

### 4.3. Procedure for Synthesis of E-2-((1H-1,2,4-Triazol-5-yl)thio)-N-benzylidene-N-phenylacetohydrazide

To a solution of 1*H*-1,2,4-Triazole-3-thiol (0.11 g, 1.0 mmol) in EtOH (10 mL), K_2_CO_3_ (0.18 g, 1.2 mmol) was added and stirred for 5 min. To this reaction mixture, **2a** (0.3 g, 1.0 mmol) was added and refluxed at 80 °C for 30 min. The progress of the reaction was monitored by TLC and cooled to RT; ice-cold water was added, and the precipitated solid was filtered and dried. The crude product was purified by column chromatography on silica gel (60–120 mesh) using a hexane-ethyl acetate solvent mixture (70:30) as the mobile phase to obtain the desired product.

### 4.4. Spectral Data of Synthesized Compounds (***4a***–***4n***)

*E-2-((1H-1,2,4-triazol-3-yl)thio)-N′-benzylidene-N-phenylacetohydrazide* (**4a**)

White solid; Yield: 93%; m.p.: 170–172 °C; ^1^H NMR (400 MHz, DMSO d_6_) δ 10.68 (s, 1H, NH), 10.08 (s, 1H, CH-triazole), 9.11 (s, 1H, N=CH), 7.84 (d, *J* = 7.0 Hz, 2H, Ar), 7.60 (d, *J* = 6.9 Hz, 2H, Ar), 7.28–7.39 (m, 4H, Ar), 7.10 (d, *J* = 7.0 Hz, 2H, Ar), 4.04 (s, 2H, CH_2_). ^13^C NMR (100 MHz, DMSO d_6_) δ 167.11, 160.89, 158.73, 139.23, 138.06, 129.24, 129.17, 125.01, 123.85, 120.93, 120.85, 119.71, 43.48; HRMS (ESI): C_17_H_15_N_5_OS [M+H]^+^ Calculated: 338.1075; Found: 338.1078.

*E-2-((1H-1,2,4-triazol-3-yl)thio)-N′-(2-chlorobenzylidene)-N-phenylacetohydrazide* (**4b**)

White solid; Yield: 90%; m.p.: 120–122 °C; ^1^H NMR (400 MHz, DMSO d_6_) δ 14.10 (s, 1H, NH), 8.58 (s, 1H, CH-triazole), 8.05 (s, 1H, N=CH), 7.64 (t, *J* = 7.5 Hz, 2H, Ar), 7.54–7.59 (m, 2H, Ar), 7.44–7.48 (m, 3H, Ar), 7.30 (d, *J* = 7.5 Hz, 2H, Ar), 4.70 (s, 2H, CH_2_). ^13^C NMR (100 MHz, DMSO d_6_) δ 169.90, 158.91, 145.29, 137.73, 135.69, 133.65, 132.07, 131.27, 130.81, 130.39, 130.19, 129.46, 128.22, 127.60, 35.25. HRMS (ESI): C_17_H_14_^35^ClN_5_OS [M+H]^+^; Calculated: 372.0686; Found: 372.0689. C_17_H_14_^37^ClN_5_OS [M+H]^+^; Calculated: 374.0686; Found: 374.0662.

*E-2-((1H-1,2,4-triazol-3-yl)thio)-N′-(4-chlorobenzylidene)-N-phenylacetohydrazide* (**4c**)

White solid; Yield: 90%; m.p.: 174–176 °C; ^1^H NMR (400 MHz, DMSO d_6_) δ 14.09 (s, 1H, NH), 8.58 (s, 1H, CH-triazole), 8.03 (s, 1H, N=CH), 7.62–7.66 (m, 3H, Ar), 7.49–7.59 (m, 4H, Ar), 7.29 (d, *J* = 7.5 Hz, 2H, Ar), 4.69 (s, 2H, CH_2_). ^13^C NMR (100 MHz, DMSO) δ 169.93, 158.85, 145.29, 135.66, 134.33, 130.83, 130.40, 130.24, 129.86, 129.41, 128.82, 128.57, 35.15. HRMS (ESI): C_17_H_14_^35^ClN_5_OS [M+H]^+^; Calculated: 372.0686; Found: 372.0689. C_17_H_14_^37^ClN_5_OS [M+H]^+^; Calculated: 374.0686; Found: 374.0659.

*E-2-((1H-1,2,4-triazol-3-yl)thio)-N′-(4-fluorobenzylidene)-N-phenylacetohydrazide* (**4d**)

White solid; Yield: 89%; m.p.: 174–176 °C; ^1^H NMR (400 MHz, DMSO d_6_) δ 14.08 (s, 1H, NH), 8.58 (s, 1H, CH-triazole), 7.74 (m, *J* = 8.0, 5.3 Hz, 2H, Ar), 7.60 (d, *J* = 7.8 Hz, 2H, Ar), 7.55 (d, *J* = 7.8 Hz, 1H, Ar), 7.31 (s, 1H, N=CH), 7.24–7.27 (m, 4H, Ar), 4.67 (s, 2H, CH_2_). ^13^C NMR (100 MHz, DMSO d_6_) δ 169.73, 164.77, 162.30, 159.01, 152.37, 145.27, 140.76, 135.76, 130.96 (*J* = 2.4 Hz), 130.93, 130.67, 129.91 (*J* = 8.2 Hz), 129.87, 129.62, 116.44, 116.22, 35.31. HRMS (ESI): C_17_H_14_FN_5_OS [M+H]^+^; Calculated: 356.0981; Found: 356.0982.

*E-2-((1H-1,2,4-triazol-3-yl)thio)-N′-(4-bromobenzylidene)-N-phenylacetohydrazide* (**4e**)

White solid; Yield: 92%; m.p.: 202–204 °C; ^1^H NMR (400 MHz, DMSO d_6_) δ 14.08 (s, 1H, NH), 8.58 (s, 1H, CH-triazole), 7.74 (m, *J* = 8.0, 5.3 Hz, 2H, Ar), 7.60 (d, *J* = 7.6 Hz, 2H, Ar), 7.55 (d, *J* = 7.6 Hz, 1H, Ar), 7.31 (s, 1H, N=CH), 7.24–7.27 (m, 4H, Ar), 4.67 (s, 2H, CH_2_). ^13^C NMR (100 MHz, DMSO d_6_) δ 164.77, 162.30, 159.01, 145.27, 140.76, 135.76, 130.67, 129.95 129.87, 129.62, 116.44, 116.22, 35.31. HRMS (ESI): C_17_H_14_^79^BrN_5_OS [M+H]^+^; Calculated: 416.0180; Found: 416.0184. C_17_H_14_^81^BrN_5_OS [M+H]^+^; Calculated: 418.0180; Found: 418.0164.

*E-2-((1H-1,2,4-triazol-3-yl)thio)-N′-(4-nitrobenzylidene)-N-phenylacetohydrazide* (**4f**)

White solid; Yield: 90%; m.p.: 200–202 °C; ^1^H NMR (400 MHz, DMSO d_6_) δ 14.09 (s, 1H, NH), 8.57 (s, 1H, CH-triazole), 8.26 (d, *J* = 8.8 Hz, 2H, Ar), 7.95 (d, *J* = 8.8 Hz, 2H, Ar), 7.62 (d, *J* = 7.7 Hz, 2H, Ar), 7.56 (t, *J* = 6.7 Hz, 1H, Ar), 7.43 (s, 1H, N=CH), 7.28 (d, *J* = 7.5 Hz, 2H, Ar), 4.70 (s, 2H, CH_2_). ^13^C NMR (100 MHz, DMSO d_6_) δ 170.06, 166.11, 158.85, 148.27, 145.32, 140.63, 135.56, 130.75, 130.13, 129.49, 128.68, 124.45, 35.12. HRMS (ESI): C_17_H_14_N_6_O_3_S [M+H]^+^; Calculated: 383.0926; Found: 383.0927.

*E-2-((1H-1,2,4-triazol-3-yl)thio)-N′-(2-fluorobenzylidene)-N-phenylacetohydrazide* (**4g**)

White solid; Yield: 88%; m.p.: 170–172 °C; ^1^H NMR (400 MHz, DMSO d_6_) δ 14.10 (s, 1H, NH), 8.58 (s, 1H, CH-triazole), 8.05 (s, 1H, N=CH), 7.64 (t, *J* = 7.5 Hz, 2H, Ar), 7.54–7.59 (m, 2H, Ar), 7.44–7.48 (m, 3H, Ar), 7.30 (d, *J* = 7.5 Hz, 2H, Ar), 4.70 (s, 2H, CH_2_). ^13^C NMR (100 MHz, DMSO d_6_) δ 169.92, 164.77, 162.30, 156.97, 146.95, 140.82, 135.74, 130.95 (*J* = 3.0 Hz), 130.92, 130.66, 129.98, 129.92, 129.89, 129.63, 116.43, 116.21, 35.59. HRMS (ESI): C_17_H_14_FN_5_OS [M+H]^+^; Calculated: 356.0981; Found: 356.0984.

*E-2-((1H-1,2,4-triazol-3-yl)thio)-N′-(3-bromobenzylidene)-N-phenylacetohydrazide* (**4h**)

White solid; Yield: 90%; m.p.: 160–162 °C; ^1^H NMR (400 MHz, DMSO d_6_) δ 14.09 (s, 1H, NH), 8.57 (s, 1H, CH-triazole), 7.91 (d, *J* = 6.6 Hz, 1H, Ar), 7.68 (d, *J* = 7.8 Hz,1H, Ar), 7.61 (t, *J* = 7.5 Hz, 3H, Ar), 7.54 (t, *J* = 7.4 Hz, 1H, Ar), 7.37 (t, *J* = 7.7 Hz, 1H, Ar), 7.31 (s, 1H, N=CH), 7.25 (d, *J* = 7.25 Hz, 2H, Ar), 4.68 (s, 2H, CH_2_). ^13^C NMR (100 MHz, DMSO d_6_) δ 169.86, 158.96, 145.27, 140.35, 136.78, 135.65, 133.04, 131.37, 130.69, 130.15, 129.99, 129.56, 126.65, 122.62, 35.26. HRMS (ESI): C_17_H_14_^79^BrNOS [M+H]^+^; Calculated: 416.0180; Found: 416.0181. C_17_H_14_^81^BrNOS [M+H]^+^; Calculated: 418.0180; Found: 418.0161.

*E-2-((1H-1,2,4-triazol-3-yl)thio)-N′-(2,4-dichlorobenzylidene)-N-phenylacetohydrazide* (**4i**)

White solid; Yield: 91%; m.p.: 160–162 °C; ^1^H NMR (400 MHz, DMSO d_6_) δ 14.08 (s, 1H, NH), 8.58 (s, 1H, CH-triazole), 7.70 (d, *J* = 8.3 Hz, 2H, Ar), 7.61 (t, *J* = 7.4 Hz, 2H, Ar), 7.54 (t, *J* = 7.4 Hz, 1H, Ar), 7.48 (d, *J* = 8.5 Hz, 2H, Ar), 7.30 (s, 1H, N=CH), 7.26 (d, *J* = 8.4 Hz, 2H, Ar), 4.67 (s, 2H, CH_2_). ^13^C NMR (100 MHz, DMSO d_6_) δ 169.79, 158.96, 156.85, 145.28, 145.11, 140.68, 140.63, 135.66, 135.01, 133.25, 130.69, 129.99, 129.60, 129.35, 35.26. HRMS (ESI): C_17_H_13_^35^Cl_2_N_5_OS [M+H]^+^; Calculated: 406.0296; Found: 406.0298. C_17_H_13_^37^Cl_2_N_5_OS [M+H]^+^; Calculated: 406.0296; Found: 406.0298.

*E-2-((1H-1,2,4-triazol-3-yl)thio)-N′-(2,6-dichlorobenzylidene)-N-phenylacetohydrazide* (**4j**)

White solid; Yield: 90%; m.p.: 163–165 °C; ^1^H NMR (400 MHz, DMSO d_6_) δ 14.07 (s, 1H, NH), 8.56 (s, 1H, CH-triazole), 7.64 (d, *J* = 7.6 Hz, 2H, Ar), 7.54 (d, *J* = 8.2 Hz, 3H, Ar), 7.48 (s, 1H, N=CH), 7.40–7.44 (m, 1H, Ar), 7.31 (d, *J* = 7.5 Hz, 2H, Ar), 4.67 (s, 2H, CH_2_). ^13^C NMR (100 MHz, DMSO d_6_) δ 169.91, 158.91, 152.33, 145.20, 137.38, 135.28, 134.41, 131.73, 130.81, 130.21, 129.85, 129.52, 35.53. HRMS (ESI): C_17_H_13_^35^Cl_2_N_5_OS [M+H]^+^; Calculated: 406.0296; Found: 406.0298. C_17_H_13_^37^Cl_2_N_5_OS [M+H]^+^; Calculated: 408.0296; Found: 408.0266.

*E-2-((1H-1,2,4-triazol-3-yl)thio)-N′-(4-methoxybenzylidene)-N-phenylacetohydrazide* (**4k**)

White solid; Yield: 90%; m.p.: 194–196 °C; ^1^H NMR (400 MHz, DMSO d_6_) δ 14.07 (s, 1H, NH), 8.57 (s, 1H, CH-triazole), 7.58–7.62 (m, 4H, Ar), 7.53 (t, *J* = 6.8 Hz, 1H, Ar), 7.25 (s, 1H, N=CH), 7.24 (s, 2H, Ar), 6.97 (d, *J* = 8.5 Hz, 2H, Ar), 4.65 (s, 2H, CH_2_), 3.78 (s, 3H, OCH_3_). ^13^C NMR (100 MHz, DMSO d_6_) δ 169.52, 161.28, 159.10, 145.25, 141.82, 135.89, 130.63, 129.82, 129.70, 129.34, 126.86, 114.76, 55.76, 35.40. HRMS (ESI): C_18_H_17_N_5_O_2_S [M+H]^+^; Calculated: 368.1181; Found: 368.1183.

*E-2-((1H-1,2,4-triazol-3-yl)thio)-N-phenyl-N′-(3,4,5-trimethoxybenzylidene)acetohydrazide* (**4l**)

White solid; Yield: 90%; m.p.: 122–124 °C; ^1^H NMR (400 MHz, DMSO) δ 14.07 (s, 1H, NH), 8.56 (s, 1H, CH-triazole), 7.61 (d, *J* = 7.7 Hz, 2H, Ar), 7.54 (s, 1H, Ar), 7.25 (s, 2H, Ar), 7.24 (s, 1H, N=CH), 7.04 (s, 2H, Ar), 4.67 (s, 2H, CH_2_), 3.78 (s, 6H, 2*OCH_3_), 3.68 (s, 3H, OCH_3_). ^13^C NMR (100 MHz, DMSO d_6_) δ 171.25, 159.07, 153.56, 145.24, 139.54, 135.84, 130.70, 129.60, 129.60, 129.41, 122.81, 105.02, 60.59, 56.34, 35.32. HRMS (ESI): C_20_H_21_N_5_O_4_S [M+H]^+^; Calculated: 428.1392; Found: 428.1396.

*E-2-((1H-1,2,4-triazol-3-yl)thio)-N′-(4-(dimethylamino)benzylidene)-N-phenylacetohydrazide* (**4m**)

White solid; Yield: 89%; m.p.: 176–178 °C; ^1^H NMR (400 MHz, DMSO d_6_) δ 14.07 (s, 1H, NH), 8.58 (s, 1H, CH-triazole), 7.59 (t, *J* = 7.5 Hz, 2H, Ar), 7.51 (t, *J* = 7.4 Hz, 1H, Ar), 7.46 (d, *J* = 8.5 Hz, 2H, Ar), 7.24 (d, *J* = 8.5 Hz, 2H, Ar), 7.17 (s, 1H, N=CH), 6.70 (d, *J* = 8.7 Hz, 2H, Ar), 4.68 (d, 2H, CH_2_), 3.34 (s, 6H, 2*CH_3_). ^13^C NMR (100 MHz, DMSO d_6_) δ 169.18, 159.22, 151.95, 145.23, 142.85, 136.12, 130.56, 129.75, 129.67, 129.08, 121.52, 112.17, 40.61, 35.51. HRMS (ESI): C_19_H_20_N_6_OS [M+H]^+^; Calculated: 381.1497; Found: 381.1497.

*E-2-((1H-1,2,4-triazol-3-yl)thio)-N-phenyl-N′-(thiophen-2-ylmethylene)acetohydrazide* (**4n**)

White solid; Yield: 88%; m.p.: 208–210 °C; ^1^H NMR (400 MHz, DMSO d_6_) δ 14.08 (s, 1H, NH), 8.57 (s, 1H, CH-triazole), 7.63–7.65 (m, 1H, Ar), 7.60 (d, *J* = 7.7 Hz, 2H, Ar), 7.54 (d, *J* = 7.3 Hz, 1H, Ar), 7.50 (s, 1H, N=CH), 7.36 (d, *J* = 3.4 Hz, 1H, Ar), 7.27 (d, *J* = 7.4 Hz, 2H, Ar), 7.07 (m, *J* = 4.9, 3.8 Hz, 1H, Ar), 4.59 (s, 2H, CH_2_). ^13^C NMR (100 MHz, DMSO d_6_) δ 169.26, 159.04, 152.36, 145.24, 139.21, 137.13, 135.75, 131.76, 130.71, 129.98, 129.61, 128.40, 35.27. HRMS (ESI): C_15_H_13_N_5_OS_2_ [M+H]^+^; Calculated: 344.0640; Found: 344.0641.

### 4.5. In Silico Analysis (ADME)

The first step was to generate the SMILE (Simplified Molecular Input Line Entry System) information of the compounds using the SwissADME web-based online tool (http://www.swissadme.ch/ accessed on 3 March 2025). The second step involved the estimation of their ADMET (absorption, distribution, metabolism, excretion, and toxicity) properties using the same tool [51].

### 4.6. Solubility Optimization and Antibacterial Activity Evaluation

During screening of the novel triazole-based molecules for antibacterial activity, the molecules were not soluble in LB broth and precipitated at higher concentrations. Dimethyl sulfoxide (DMSO) was thus first employed as a solvent to dissolve the molecules due to its thoroughly documented nature of dissolving hydrophobic molecules without having any significant impacts on the growth of bacteria at low concentrations. For screening antibacterial activities, stock solutions were made by dissolving the compounds in DMSO (5 mg/mL) and serial dilution in LB broth till 2, 1, 0.5, 0.25, and 0.125 mg/mL, respectively. Precipitation was seen, which may have deterred antimicrobial assessment. Standardization also revealed that the compounds were completely soluble up to 0.5 mg/mL in LB broth for effective antibacterial screening. This optimized concentration was used in the following assays to explore the antibacterial activity of the synthesized compounds.

### 4.7. Bacterial Strains and Culture Conditions

Both Gram-positive and Gram-negative bacterial strains were employed in the experiment to assess antibacterial activities of the synthesized compounds. Gram-negative *Klebsiella aerogenes* ATCC 13048 and Gram-positive *Enterococcus* species MVBP-18 strains were used for the study. Bacterial strains of both were cultured in Luria–Bertani (LB) broth for optimal growth conditions. Bacterial cultures were sustained by culturing a fresh colony from newly streaked LB agar plates to 5 mL of LB medium and incubated at 37 °C with shaking at 200 rpm continuously for 16–18 h in order to achieve the logarithmic growth phase. Overnight cultures, after incubation, were standardized by dilution to an OD600 of 0.05, which contained equal bacterial inoculum for any experimental condition. These optimized cultures were employed for subsequent antibacterial tests, such as growth kinetics analysis, to determine the impact of the test compound on bacterial growth.

### 4.8. Growth Kinetics Assay

A preliminary screening was conducted in a 96-well microplate format. Standardized bacterial cultures were treated with the compounds at a final concentration of 0.5 mg/mL. Control wells included untreated bacteria and a DMSO control. The plate was incubated at 37°C, with shaking at 200 rpm continuously, and bacterial growth was monitored by measuring the OD_600_ every hour for six hours using a microplate reader ( BioTek Synergy H1). The assay was performed in triplicate.

### 4.9. CFU Enumeration

Compounds that showed significant activity in the preliminary screen were further validated. Bacterial growth was monitored by both the OD_600_ readings over 4–7 h and colony-forming unit (CFU) enumeration [52,53]. For CFU counting, aliquots from the wells were serially diluted, plated on LB agar, and incubated overnight at 37 °C. The resulting colonies were counted to determine the CFU/mL. All validation experiments were also conducted in triplicate [54,55].

### 4.10. Cytotoxicity Assessment Using MTT Assay 

Cytotoxicity was evaluated on HEK (human embryonic kidney) cell lines using the MTT assay [56]. The assay was performed on compounds that showed significant antibacterial activity and three that did not. Cells were grown in a medium that was supplemented with 10% FBS and 1% penicillin–streptomycin. This was incubated at 37 °C in a 5% CO_2_ atmosphere for 24 h. Cells were seeded at 1 × 10^3^ cells/well in 96-well plates and allowed to adhere overnight. They were then treated with the test compounds, dissolved in DMSO, at a uniform concentration. The final DMSO concentration in the culture medium did not exceed 0.5%. The control wells contained untreated cells or cells treated with DMSO alone. After 24 h of treatment, 10 µL of the MTT solution (5 mg/mL) was added to each well and incubated for 3 h to allow formazan crystal formation. A solubilization solution (100 µL) was then added to dissolve the crystals. The absorbance at 570 nm was measured using a microplate reader. Cell viability was calculated as a percentage relative to the untreated control. All assays were performed in triplicate [57].

## 5. Conclusions

In this study, a series of novel 2-((1*H*-1,2,4-triazol-5-yl)thio)-*N*-benzylidene-*N*-arylacetohydrazide hybrids (**4a**–**4n**) were designed, synthesized, and evaluated. A combined in silico and experimental approach successfully identified **4g** as a highly promising lead compound. It demonstrated potent, broad-spectrum antibacterial activity against both Gram-positive and Gram-negative bacteria. Furthermore, it exhibited less harm to a normal kidney cell line, suggesting potential for antibacterial therapies. This study also highlights the potential of this class of compounds for environmental applications in the fight against the spread of antimicrobial resistance.

Although the work points out **4g** as a highly promising 2-((1*H*-1,2,4-triazol-5-yl)thio)-*N*-benzylidene-*N*-arylacetohydrazide hybrid, its precise mechanism of action is not known, and in the absence of being aided by mechanistic data, long-term efficacy and the risk of development of resistance cannot be accurately estimated. Toxicity was tested against only a single cell line of the kidney, and hence, there is a need for broader safety tests on various types of mammalian cells before validating in the in vivo mouse models. Thus, future work of this compound involves studying the mechanism of action, resistance, and broader safety experiments, in order to achieve the full promise of these compounds.

Overall, these compounds, and particularly 4g, represent a promising scaffold for the future development of new antimicrobial agents to address critical needs in both clinical medicine and environmental health.

## Figures and Tables

**Table 1:** Optimization of reaction conditions for target molecules.

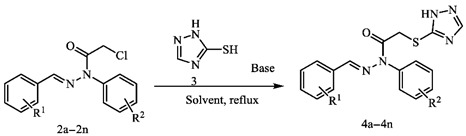					
Entry	Solvents	Bases	Time (h)	Temp (°C)	Yield (%)
1	DCM	K_2_CO_3_	3	RT	60
2	THF	K_2_CO_3_	3	60	65
3	1,4 Dioxane	K_2_CO_3_	3	80	68
4	ACN	K_2_CO_3_	2	80	74
5	Toluene	K_2_CO_3_	3	100	55
6	DMF	K_2_CO_3_	1.5	100	75
7	DMSO	K_2_CO_3_	1.5	100	78
8	IPA	K_2_CO_3_	1	80	80
9	MeOH	K_2_CO_3_	1	60	84
10	EtOH	NaHCO_3_	0.75	80	83
11	EtOH	Na_2_CO_3_	0.75	80	80
12	EtOH	K*^t^*OBu	1	80	72
13	EtOH	K_2_CO_3_	0.25	80	90
14	EtOH	TEA	2	80	65
15	EtOH	DIPEA	2	80	68
16	EtOH	K_2_CO_3_	2	RT	80
17	EtOH	K_2_CO_3_	1	50	82

**Table 2:** Synthesized 2-((1*H*-1,2,4-triazol-5-yl)thio)-*N*-substituted benzylidene-*N*-phenylacetohydrazide derivatives.

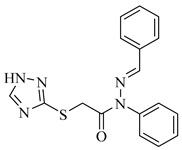	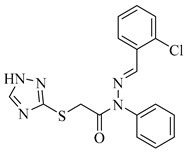	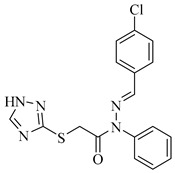
**4a** 93%	**4b** 90%	**4c** 90%
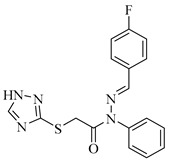	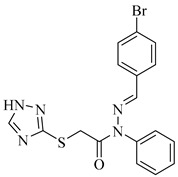	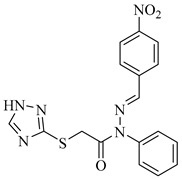
**4d** 89%	**4e** 92%	**4f** 90%
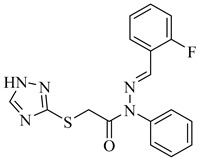	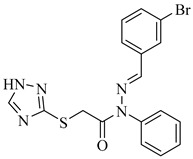	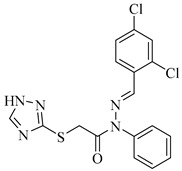
**4g** 88%	**4h** 90%	**4i** 91%
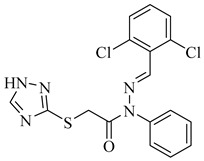	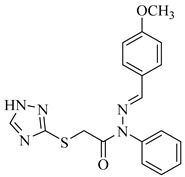	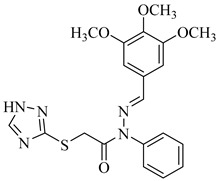
**4j** 90%	**4k** 90%	**4l** 90%
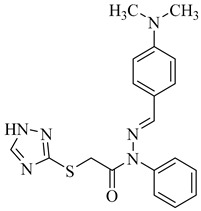	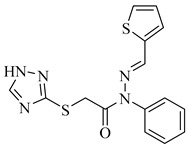	
**4m** 89%	**4n** 88%	

*N′*-substituted benzylidene-2-chloro-*N*-phenylacetohydrazide (1 mmol), 1*H*-1,2,4-triazole-5-thiol (1 mmol), K_2_CO_3_ (1.2 mmol), EtOH (10 mL), reflux.

## Data Availability

The original contributions presented in this study are included in the article/supplementary material. Further inquiries can be directed to the corresponding authors.

## Supplementary Materials

The following supporting information can be downloaded at: https://amritauniv-my.sharepoint.com/:f:/g/personal/am_ls_r4bio24433_am_students_amrita_edu/EkDTNSTDlIFJmu5IUaAum_kB2rqAfXfEV9H9uIIXaq_gLg?e=uIDzcQ which was created on 21 September 2025.

## Abbreviations

The following abbreviations are used in this manuscript:

AMR	Antimicrobial Resistance
CFU	Colony Forming Units
DMSO	Dimethyl sulfoxide
HEK	Human Embryonic Kidney
NCEs	New Chemical Entities
DMF	Dimethylformamide
TEA	Triethylamine
DIPEA	Diisopropylethylamine
H NMR	Hydrogen-1 Nuclear Magnetic Resonance
C NMR	Carbon-13 Nuclear Magnetic Resonance
ESI MS	Electrospray Ionization Mass Spectrometry
SAR	Structure-Activity Relationship
ADMET	Absorption, Distribution, Metabolism, Excretion and Toxicity
MTT	3-(4,5-dimethylthiazol-2-yl)-2,5-diphenyltetrazolium bromide
MIC	Minimum Inhibitory Concentration
LB	Luria Bertani broth
SEM	Standard Error of the Mean
